# Cultivation-Independent Analysis of the Bacterial Community Associated With the Calcareous Sponge *Clathrina clathrus* and Isolation of *Poriferisphaera corsica* Gen. Nov., Sp. Nov., Belonging to the Barely Studied Class *Phycisphaerae* in the Phylum *Planctomycetes*

**DOI:** 10.3389/fmicb.2020.602250

**Published:** 2020-12-22

**Authors:** Nicolai Kallscheuer, Sandra Wiegand, Timo Kohn, Christian Boedeker, Olga Jeske, Patrick Rast, Ralph-Walter Müller, Franz Brümmer, Anja Heuer, Mike S. M. Jetten, Manfred Rohde, Mareike Jogler, Christian Jogler

**Affiliations:** ^1^ Department of Microbiology, Radboud University, Nijmegen, Netherlands; ^2^ Institute for Biological Interfaces 5, Karlsruhe Institute of Technology, Eggenstein-Leopoldshafen, Germany; ^3^ Leibniz Institute DSMZ, Braunschweig, Germany; ^4^ Faculty for Energy-, Process- and Bioengineering, University of Stuttgart, Stuttgart, Germany; ^5^ Institute of Biomaterials and Biomolecular Systems, University of Stuttgart, Stuttgart, Germany; ^6^ Central Facility for Microscopy, Helmholtz Centre for Infection Research, Braunschweig, Germany; ^7^ Department of Microbial Interactions, Institute of Microbiology, Friedrich Schiller University, Jena, Germany

**Keywords:** marine bacteria, sponge, 16S rRNA gene, planctomycetes, Mediterranean Sea, bacterial community

## Abstract

Marine ecosystems serve as global carbon sinks and nutrient source or breeding ground for aquatic animals. Sponges are ancient parts of these important ecosystems and can be found in caves, the deep-sea, clear waters, or more turbid environments. Here, we studied the bacterial community composition of the calcareous sponge *Clathrina clathrus* sampled close to the island Corsica in the Mediterranean Sea with an emphasis on planctomycetes. We show that the phylum *Planctomycetes* accounts for 9% of the *C. clathrus*-associated bacterial community, a 5-fold enrichment compared to the surrounding seawater. Indeed, the use of *C. clathrus* as a yet untapped source of novel planctomycetal strains led to the isolation of strain KS4^T^. The strain represents a novel genus and species within the class *Phycisphaerae* in the phylum *Planctomycetes* and displays interesting cell biological features, such as formation of outer membrane vesicles and an unexpected mode of cell division.

## Introduction

Aquatic ecosystems comprise biological communities of organisms, which together shape a local habitat. Such ecosystems include kelp forests, seagrass meadows, salt marshes, and coral reefs, which are occupied by photosynthetic primary producers, animals, and microbial communities that live in interaction with each other (Foster and Schiel, 1985; Häder et al., 1998). Sponges are ubiquitous, ancient and environmentally important members of such marine ecosystems. They are common in clear waters of tropic reefs, such as the Caribbean, but can also be found in caves, the deep-sea, and more turbid environments (Hooper and van Soest, 2002). Approximately 5% of the over 8,000 known species of the phylum *Porifera* are calcareous sponges (class *Calcarea*), which prefer shallow water and are often hidden in cave areas. Species of this class have a calcareous skeletal network (instead of a siliceous skeleton) and lack chitin (Ruys, 2013; Żółtowska, 2019). With a special interest in the relevance of the bacterial phylum *Planctomycetes*, here we analyzed the bacterial community composition on the surface of the calcareous sponge *Clathrina clathrus* Schmidt, 1864, which has a yellow color, reaches sizes of up to 10 cm, and inhabits the Atlantic coast of Western Europe and coasts in the Mediterranean Sea (Burton, 1963; Hooper and van Soest, 2002). Previous cultivation-dependent analyses of bacterial communities associated with *C. clathrus* showed that *α*- and *γ*-proteobacteria may account for up to 75–90% of the phylogenetic bacterial diversity (Muscholl-Silberhorn et al., 2008; Roué et al., 2012). The residual 10–25% were attributed to *Firmicutes* and *Actinobacteria*. However, although not previously found as dominant members in *C. clathrus*-associated bacterial communities, strains belonging to the phylum *Planctomycetes* are frequently isolated from marine and freshwater sponges (Pimentel-Elardo et al., 2003; Gade et al., 2004; Izumi et al., 2013; Kohn et al., 2020b). Members of *Planctomycetia*, the class within the phylum *Planctomycetes* with the currently highest number of described species, are often found attached to biotic and abiotic marine surfaces, on which they can be highly abundant (Lage and Bondoso, 2014; Wiegand et al., 2018). Once attached to a biotic surface, e.g., a macroscopic phototroph, planctomycetes are thought to use released polysaccharides as carbon and energy source probably using a specialized system for uptake of entire high-molecular weight sugar molecules and subsequent intracellular degradation (Boedeker et al., 2017). Employing such a system represents a decisive advantage compared to other, often faster growing bacteria inhabiting the same ecological niche, e.g., members of the “*Roseobacter* group” (Frank et al., 2015). Many of such competitors use catabolic exoenzymes for an extracellular cleavage of polysaccharides, a strategy which provides easily available carbon sources also to other members of the bacterial community. Other planctomycetal characteristics, such as the potential for production of secondary metabolites or the observed resistance to several antibiotics, might also contribute to the compensation of lower growth rates of planctomycetes (Jeske et al., 2013; Godinho et al., 2019) and are worth to be investigated in more detail.

**Figure 1:**
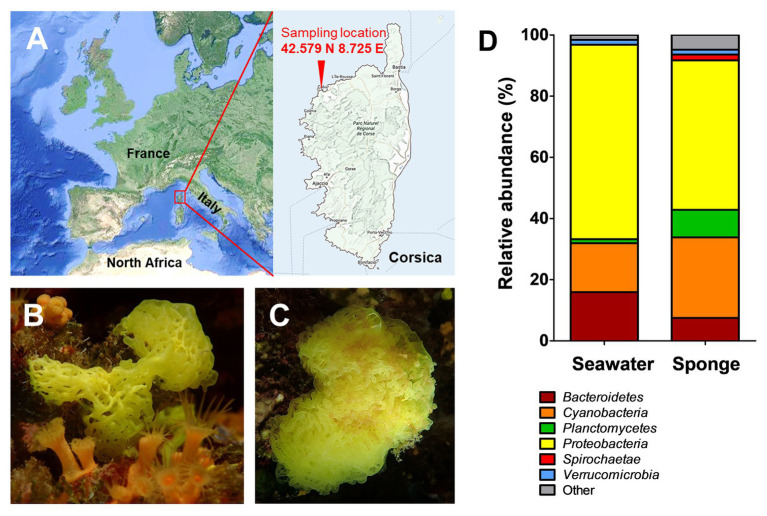
Sampling location and results of the cultivation-independent analysis of the *Clathrina clathrus*-associated bacterial community. Samples were taken close to Calvi on the Northern coast of the island Corsica, France (**A**; exact coordinates are given in the picture). Picture of the investigated calcareous sponge as side view **(B)** and top view **(C)**. Determination of the bacterial community composition of *C. clathrus* on bacterial phylum level compared to surrounding seawater **(D)**. Data for the surrounding seawater was taken from a previous study (Kohn et al., 2020a).

Well-established methods, however, can often not be applied for planctomycetes without extensive optimization. Members of this phylum likely escaped detection due to the use of mismatching 16S rRNA gene primers and due to cultivation bias discriminating against slow-growing bacteria (for review see Wiegand et al., 2018). This can provide an explanation why they have not been captured in previous cultivation-dependent studies. A similar argumentation might be applicable for a previous analysis of a *C. clathrus*-associated bacterial community, in which *γ*- and *α*-*Proteobacteria* turned out to be the bacterial classes with the highest relative abundances of 83 and 14%, respectively (Quévrain et al., 2014). Similar observations were made for the related calcareous sponge *Leuconia johnstoni*, for which the relative abundance of *γ*-*Proteobacteria* was slightly lower (61%), but instead a higher abundance of *Bacteroidetes* (12%) and *Firmicutes* (9%) was determined (Quévrain et al., 2014). In the above-mentioned study, the authors followed a cultivation-dependent approach and incubated enriched bacterial cell suspensions extracted from calcareous sponges on nutrient-rich marine agar plates. Plates were cultivated for only 2 days at a relatively low temperature of 18°C. These conditions are not suitable for the isolation of planctomycetes, which have generation times ranging from 8 h to several days and typically show optimal growth at temperatures of 26–30°C (Wiegand et al., 2018). In addition to different optimal conditions of bacterial species inhabiting such communities, e.g., with regard to temperature, pH, and NaCl concentration, differences in growth rates inevitably lead to biased abundance of different bacterial phyla, favoring fast-growing species well-adapted to the used cultivation medium. A related study showed that members of the phylum *Spirochaetes* can be present in considerable numbers in *C. clathrus*-associated communities, but escaped previously performed analyses (Neulinger et al., 2010). The recent development of novel tools and laboratory techniques motivated us to reinvestigate calcareous sponges for the occurrence of planctomycetes (Jogler and Jogler, 2013; Wiegand et al., 2020). For this purpose, we included a cultivation-independent analysis of the bacterial community of *C. clathrus* sampled near the coast of the island Corsica in the Mediterranean Sea (Figures 1A–C). By using an optimized medium formulation, we were also able to isolate planctomycete strain KS4^T^, a novel member of the class *Phycisphaerae*, from *C. clathrus* and analyzed its phylogenetic position, as well as phenotypic, genomic and genome-encoded features. The description of strain KS4^T^ and access to an axenic culture are prerequisite to further investigate the barely studied class *Phycisphaerae* in the phylum *Planctomycetes*. In the long run, the large observed differences in the natural abundance of planctomycetes on different marine biotic surfaces (ranging from <1 to over 80%) can be analyzed based on presented sequencing data in this and closely related studies.

## Materials and Methods

### Cultivation-Independent Bacterial Community Analysis

The calcareous sponge *C. clathrus* was sampled in September 2015 near the coast of the island Corsica (exact location: 42.579° N 8.725° E; Figure 1A). One individual was sampled, and three pieces were used for DNA extraction and pooled for further processing. The sample for DNA extraction from the sponge surface was obtained by scraping off the sponge-associated biofilm from the three pieces using single-use scalpels and resuspension of the pooled material into sterile natural seawater. The surrounding seawater was sampled at three different locations in the immediate vicinity of the sponge and further processed within 8 h. In the laboratory, the triplicate water samples (approximately 300 ml each) were homogenized by gentle stirring and subsequently filtered through a single polycarbonate membrane filter (Ø 47 mm, Isopore, Merck) with a pore size of 0.22 μm to collect planktonic bacteria. Filters were frozen and stored at −20°C until DNA extraction. Isolation of DNA and all subsequent steps for amplification of DNA and amplicon sequencing were performed as previously described (Kohn et al., 2020a). The analysis of operational taxonomic units present in the samples was performed using the SILVAngs pipeline (Quast et al., 2012). The analysis was based on 22,364 sequences of the variable V3 region of 16S rRNA genes with an average length of 143 bp. Classification of the sequences was performed using SILVA reference version 132 and BLASTn version 2.2.30+ with a sequence similarity of 93%.

### Isolation of Strain KS4^T^ and Physiological Characterization

For the isolation of strain KS4^T^, a small piece of *C. clathrus* was washed with sterile-filtered seawater, additionally supplemented with 100 mg/L cycloheximide to prevent fungal growth. Afterward, the material was swabbed over three M1H NAG ASW plates solidified with 8 g/L gellan gum and supplemented with 1,000 mg/L streptomycin, 200 mg/L ampicillin, and 20 mg/L cycloheximide (Wiegand et al., 2020). Plates were subsequently incubated at 20°C for 8 weeks and regularly inspected for the presence of colonies. Slow-growing colonies with a white or pink color were subjected to colony-PCR to amplify the 16S rRNA gene, which was subsequently sequenced. Strains identified as members of the phylum *Planctomycetes*, but with a 16S rRNA gene similarity below 98% to already chracterized strains, were further cultivated. Strain KS4^T^, isolated using the above-mentioned strategy, showed good growth also in liquid M1H NAG ASW medium (Boersma et al., 2019) and was characterized in detail. Unless otherwise stated, growth experiments were performed at pH 7.5, and 27°C. For determination of the pH optimum for growth, different buffering compounds were used: 100 mM 2-(*N*-morpholino)ethanesulfonic acid (MES) for pH 5–6, 100 mM 4-(2-hydroxyethyl)-1-piperazineethanesulfonic acid (HEPES) for pH 7-8, or 100 mM *N*-cyclohexyl-2-aminoethanesulfonic acid (CHES) for pH 9–10. In the growth experiments without either NaCl (0% w/v NaCl) or artificial seawater (ASW) (0% ASW), the medium still contained all chemical elements required for growth (see medium recipe; Wiegand et al., 2020). Concentrated ASW contained 46.94 g/L NaCl, 7.84 g/L Na_2_SO_4_, 21.28 g/L MgCl_2_ x 6 H_2_O, 2.86 g/L CaCl_2_ x 2 H_2_O, 0.384 g/L NaHCO_3_, 1.384 g/L KCl, 0.192 g/L KBr, 0.052 g/L H_3_BO_3_, 0.08 g/L SrCl_2_ x 6 H_2_O, and 0.006 g/L NaF. 250 ml of concentrated ASW were used for the preparation of 1 L of M1H NAG ASW medium and was considered 100% ASW. A range of 0–200% ASW was tested in the cultivation experiments.

### Morphological Characterization

Phase contrast and field emission scanning electron microscopy were performed as previously described (Kohn et al., 2016).

### Genome Information and Analysis of Genome-Encoded Features

The genome sequence of strain KS4^T^ is available from GenBank under the accession number CP036425. The 16S rRNA gene sequence can be found under accession number MK559978. DNA isolation and genome sequencing are part of a previously published study (Wiegand et al., 2020). The number of carbohydrate-active enzymes was obtained from the CAZy database (Terrapon et al., 2017). Biosynthetic gene clusters associated to secondary metabolite production were determined using antiSMASH version 4.0 (Blin et al., 2017).

### Phylogenetic Analysis

The 16S rRNA gene sequence-based phylogeny was computed for strain KS4^T^, the type strains of all described planctomycetal species (assessed in January 2020) and all isolates recently published and described (Boersma et al., 2019; Kallscheuer et al., 2019a,b,c, 2020; Dedysh et al., 2020; Kohn et al., 2020a; Peeters et al., 2020). The 16S rRNA gene sequences were aligned with SINA (Pruesse et al., 2012), and the phylogenetic inference was calculated with RAxML (Stamatakis, 2014) using a maximum likelihood approach with 1,000 bootstraps, nucleotide substitution model GTR, gamma distributed rate variation, and estimation of proportion of invariable sites (GTRGAMMAI option). The 16S rRNA genes of the following bacterial strains from the *Planctomycetes*-*Verrucomicrobia*-*Chlamydiae* (PVC) superphylum outside of the phylum *Planctomycetes* were used as the outgroup: *Opitutus terrae* (NCBI accession number: AJ229235), *Kiritimatiella glycovorans* (NCBI accession number: NR_146840), and *Lentisphaera araneosa* (NCBI accession number: NR_027571). For the multi-locus sequence analysis (MLSA), the unique single-copy core genome of the analyzed genomes was determined with proteinortho5 (Lechner et al., 2011) with the “selfblast” option enabled. The protein sequences of the resulting orthologous groups were aligned using MUSCLE v.3.8.31 (Edgar, 2004). After clipping, partially aligned *C*- and *N*-terminal regions and poorly aligned internal regions were filtered using Gblocks (Castresana, 2000). The final alignment was concatenated and clustered using the maximum likelihood method implemented by RAxML (Stamatakis, 2014) with the “rapid bootstrap” method and 500 bootstrap replicates. Two strains from the family *Isosphaeraceae* (*Singulisphaera acidiphila*, accession number CP003364.1, and *Isosphaera pallida*, accession number CP002353.1) were used as outgroup in the MLSA-based phylogenetic tree. Average nucleotide identities (ANI) were calculated using OrthoANI (Lee et al., 2016). The average amino acid identity (AAI) was calculated using the aai.rb script of the enveomics collection (Rodriguez-R and Konstantinidis, 2016) and the percentage of conserved proteins (POCP) was calculated as described (Qin et al., 2014).

## Results and Discussion

### Cultivation-Independent Analysis of *Clathrina clathrus*-Associated Bacteria

**Figure 2:**
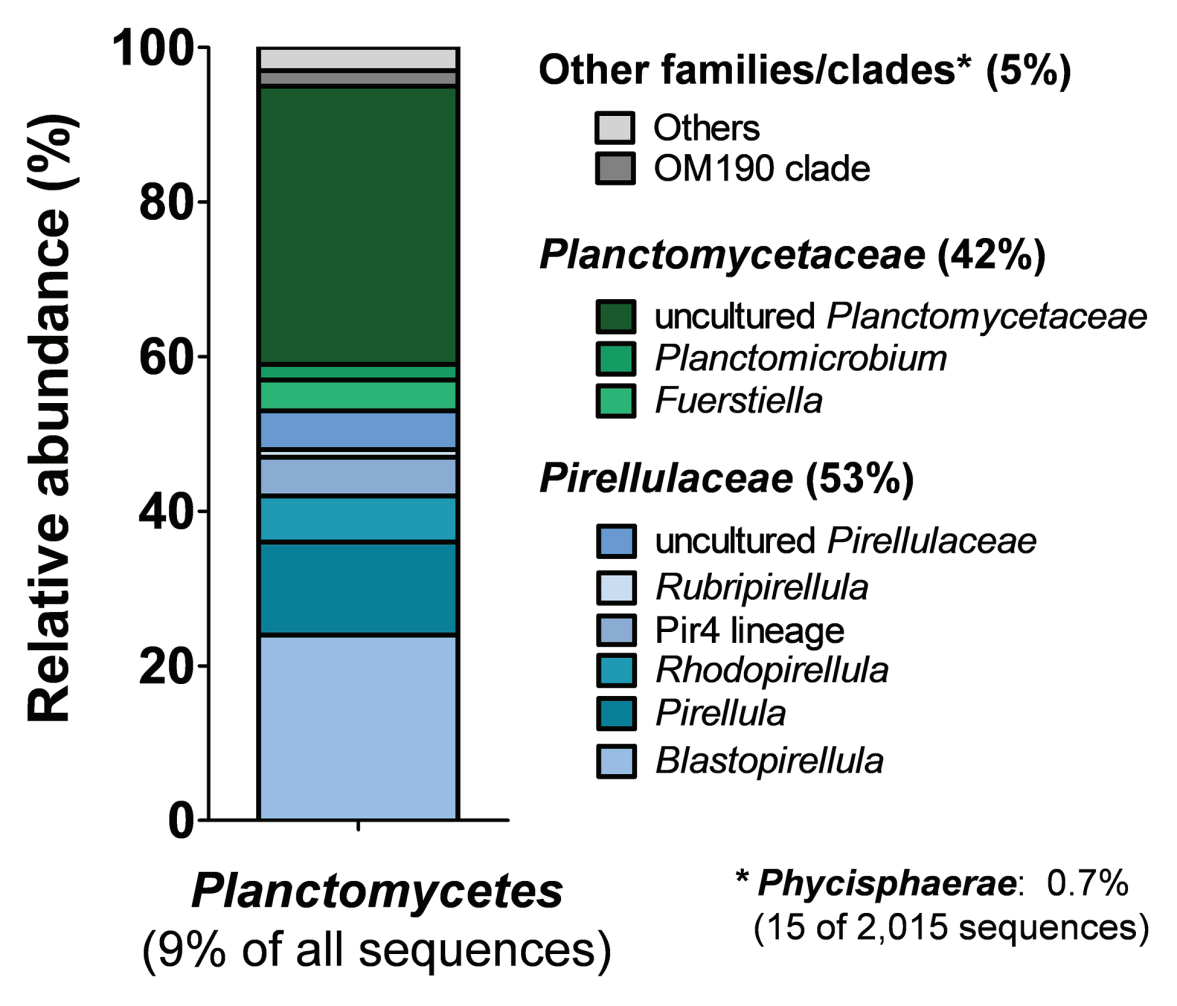
Analysis of amplicon sequences belonging to the phylum *Planctomycetes*. Of the total number of 22,364 partial 16S rRNA gene sequences, 2,015 (9.0%) belong to strains of the phylum *Planctomycetes*. Relative abundances with regard to the planctomycete-derived sequences (2,015 sequences = 100%) were further analyzed on the order/family or genus level.

In order to get an unbiased impression of the bacterial diversity associated with *C. clathrus*, we followed a cultivation-independent approach based on 16S rRNA gene amplicon sequencing. The assignment of the 16S rRNA gene sequences (in this case: the variable V3 region of the 16S rRNA) to different bacterial phyla excludes most of the influence of growth characteristics and physiological properties and ensures that slower-growing or non-cultivable species are also included. Samples from the surrounding seawater published in a previous study were analyzed for comparison (Kohn et al., 2020a). During assessment of obtained data on phylum level (Figure 1D), we could confirm previous observations that *Proteobacteria* is the most abundant phylum, both in the *C. clathrus*-associated community (relative abundance of 49%) and in the surrounding seawater (relative abundance of 64%; Quévrain et al., 2014). Our cultivation-independent analysis points toward a larger diversity as suggested by the previous approach based on cultivated isolates (Quévrain et al., 2014). The phyla *Cyanobacteria* and *Planctomycetes* showed the second and third highest relative abundances in the *C. clathrus*-associated community of 26 and 9%, respectively (Figure 1D). While relative abundances of *Cyanobacteria* and *Bacteroidetes* in the sponge-associated community and seawater did not differ more than 2-fold, members of the phylum *Planctomycetes* were preferentially found attached to the sponge (9.0% relative abundance) compared to the surrounding seawater (1.3%). This finding is in line with the observed preference of planctomycetes to attach to diverse biotic and abiotic surfaces and the tendency to grow in larger aggregates (Lage and Bondoso, 2014). This often leads to the formation of flakes or stable biofilms during laboratory-scale cultivation of axenic cultures of isolated strains (Boersma et al., 2019; Kallscheuer et al., 2020). The observed presence of *Spirochaetes* in the sponge-associated bacterial community is in line with previously published results (Neulinger et al., 2010), while this phylum was not detected in the surrounding seawater in our dataset. Other phyla, such as *Verrucomicrobia*, were equally abundant in both samples (1.6% relative abundance). When comparing the ratios of abundance in the sponge-associated community and in the surrounding seawater, ratios in the range of 1.0–2.5 were observed (in case of *Spirochaetes*, no ratio could be calculated as the abundance in seawater was zero). *Planctomycetes* stood out with a ratio of 6.9, further underlining the preference of members of this phylum to live in an attached or a eukaryote-associated state. Although much higher abundances of *Planctomycetes* of 60–85% on biotic surfaces were observed before (Bengtsson and Øvreås, 2010; Wiegand et al., 2018), values of nearly 10% found in this study are still in sharp contrast to the results of the previously published cultivation-dependent analysis (Quévrain et al., 2014), which probably did not capture *Planctomycetes* due to unsuitable cultivation conditions for strains of this phylum (Kohn et al., 2020a). This said, we analyzed the sequences obtained for members of the phylum *Planctomycetes* in more detail and calculated relative abundances on the order/family and genus level (Figure 2). Of a total number of 22,364 obtained sequences, 2,015 sequences belong to members of the phylum *Planctomycetes*. For the relative abundances shown in Figure 2 and in the text below, this number was set to 100%. The majority of obtained sequences could be assigned to two planctomycetal families, namely *Pirellulaceae* (53%) and *Planctomycetaceae* (42%), together accounting for 95% of the total number of *Planctomycetes*-derived sequences. Abundant genera in the family *Pirellulaceae* include *Blastopirellula* (24%), *Pirellula* (12%), and *Rhodopirellula* (6%). Uncultivated *Pirellulaceae* accounted for 5% of the 2,015 sequences. In contrast, uncultivated members made up the majority of the family *Planctomycetaceae* (36%) in our dataset, while members of the genera *Fuerstiella* (4%) and *Planctomicrobium* (2%) were also found (Figure 2). Both families, *Pirellulaceae* and *Planctomycetaceae*, more specifically the respective orders, *Pirellulales* and *Planctomycetales*, belong to the class *Planctomycetia*, which additionally comprises the orders *Gemmatales* and *Isosphaerales*. Five (0.25%) and seven (0.35%) sequences, respectively, could be assigned to these two orders. Fifteen sequences (0.7%) belong to members of *Phycisphaerae*, another class within the phylum *Planctomycetes*.

### Targeted Isolation of Novel Planctomycetes Associated to *C. clathrus*

Based on the results described above, we were eager to explore *C. clathrus* as a yet untapped source for novel planctomycetal strains. We used an established pipeline for processing the biological sampling material, isolation, and initial cultivation of planctomycetal strains (Wiegand et al., 2020). For isolation of novel planctomycetes, we typically exploit their inherent resistance against several antibiotics as the selection condition and prolong the incubation time to several weeks. Applying these two key factors, we were able to bring strain KS4^T^ into axenic culture and here present a detailed strain characterization of the novel isolate. To the best of our knowledge, strain KS4^T^ is the first planctomycete isolated from a calcareous sponge. The V3 region of the 16S rRNA gene sequence of strain KS4^T^ was absent from the amplicon dataset. The highest sequence similarity of the V3 region of 88% was found to an uncultivated planctomycete strain designated GMD14H07 (GenBank accession no. AY162124). The near full length 16S rRNA gene sequence identity of 85% was even lower and, thus, probably excludes a relationship on the genus or species level (genus threshold 94.5%, species threshold 98.7%; Stackebrandt and Ebers, 2006; Yarza et al., 2014).

### Phylogenetic Inference of Strain KS4^T^ Isolated From *C. clathrus*

**Figure 3:**
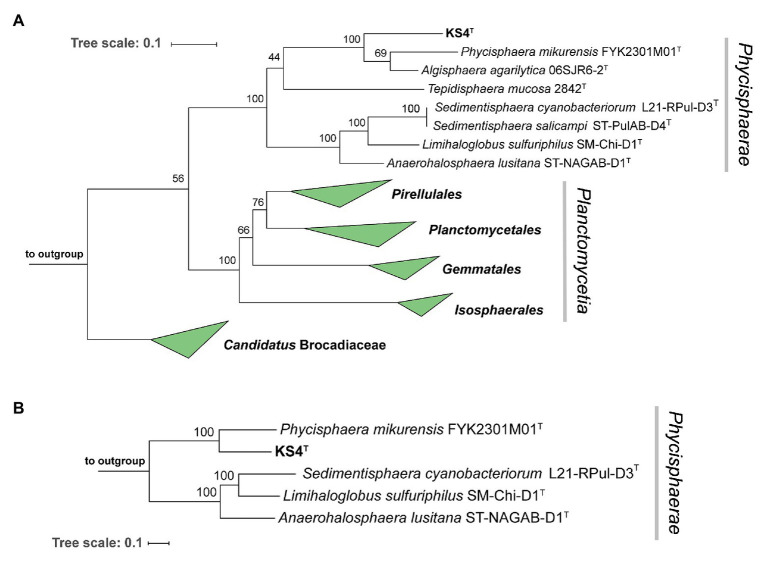
Maximum likelihood phylogenetic analysis. Phylogenetic trees showing the position of strain KS4^T^. 16S rRNA gene- **(A)** and multi-locus sequence analysis (MLSA)-based phylogenetic analysis **(B)** was performed as described in the Material and Methods section. Bootstrap values after 1,000 re-samplings (16S rRNA gene) or 500 re-samplings (MLSA) are given at the nodes (in %). The strains used as outgroup are provided in the Material and Methods section.

In phylogenetic trees based on 16S rRNA gene sequences and MLSA, strain KS4^T^ clusters within the class *Phycisphaerae* in the phylum *Planctomycetes* (Fukunaga et al., 2009; Figure 3). Comparison on the level of 16S rRNA gene sequences suggests *Algisphaera agarilytica* 06SJR6-2^T^ as the current closest relative (Yoon et al., 2014), since this strain showed the highest 16S rRNA gene sequence similarity of 88.7% with strain KS4^T^. The second closest relative is *Phycisphaera mikurensis* (84.6% 16S rRNA gene sequence similarity; Fukunaga et al., 2009). Based on these values, strain KS4^T^ is likely a member of the family *Phycisphaeraceae*, order *Phycisphaerales*, class *Phycisphaerae*. 16S rRNA gene sequence similarities of strain KS4^T^ with known members of the family are below the proposed genus threshold of 94.5% (Yarza et al., 2014), which delineates strain KS4^T^ from the two described genera in the family (Figure 3A). As no genome sequence of *A. agarilytica* is available, the whole genome-based phylogenetic analysis only allowed comparison to *P. mikurensis*. The classification of strain KS4^T^ as a member of a novel genus is supported by AAI values of 44.9% and POCP of 30.9%, both clearly below the genus thresholds of 60 and 50%, respectively (Konstantinidis and Tiedje, 2005; Qin et al., 2014). An ANI of 66.8% ensures that the novel strain does not belong to the species *P. mikurensis* (species threshold 95%; Kim et al., 2014). Taken together, all analyzed phylogenetic markers support the delineation of strain KS4^T^ from the genus *Phycisphaera*. Due to the lack of genome information, delineation from the genus *Algisphaera* can only be performed based on the 16S rRNA gene comparison. However, as the observed 16S rRNA similarity of 88.0% is closer to the family threshold (86.5%) than to the genus threshold (94.5%; Yarza et al., 2014), it appears highly unlikely that strain KS4^T^ is a member of the genus *Algisphaera*.

### Morphological Features of Strain KS4^T^

**Figure 4:**
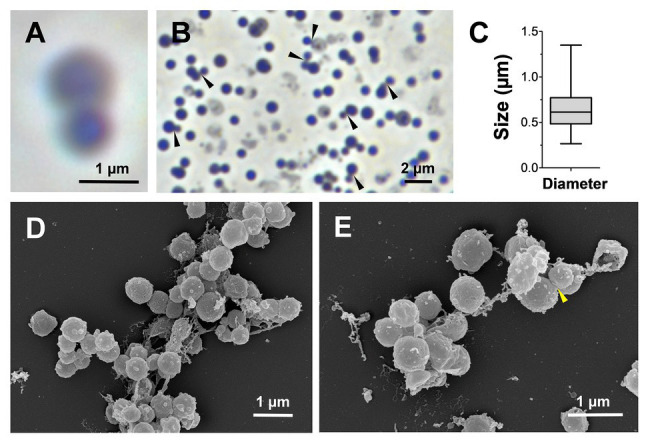
Microscopy images and cell size plot of strain KS4^T^. Phase contrast **(A,B)** and electron microscopic images **(D,E)** of strain KS4^T^ are shown. For determination of the cell size **(C)**, at least 100 representative cells were counted manually or by using a semi-automated object count tool. Cells, for which we observed budding as potential mode of cell division, are indicated by black or yellow arrows (**B** and **E**, respectively).

For a morphological characterization, cells of strain KS4^T^ harvested during the exponential growth phase were subjected to light and electron microscopy. KS4^T^ cells are rather small and spherical (average diameter of 0.6 ± 0.2 μm) and grow in larger aggregates (Figures 4A–C). Cells are motile and form white colonies on solid medium. The cell surface is rough and typically covered with conspicuous outer membrane vesicles, which were not described for closely related species (Fukunaga et al., 2009; Yoon et al., 2014; Figures 4D,E). Typical characteristics of many planctomycetes, such as the crateriform structures, the stalk structure and the holdfast structure, were not observed for strain KS4^T^. This, however, is in line with its phylogenetic position since known members of the class *Phycisphaerae* also lack a stalk or holdfast structure (Fukunaga et al., 2009; Kovaleva et al., 2015; Wiegand et al., 2018). Light and electron microscopic images indicate that strain KS4^T^ divides by budding (Figures 4A,B,E). At the final stage of cell division, in which the spherical mother and daughter cells have reached the same size, we were not able to differentiate between budding and binary fission. However, at the initial stage of cell division, large differences in the cell sizes of the mother and daughter cells are clearly observable during light microscopy (Figure 4B), which is a clear hint towards asymmetric division by budding. All known species of the class *Planctomycetia* divide by budding, however, our observation is surprising, given that strain KS4^T^ is a member of the class *Phycisphaerae*, in which all characterized species divide by binary fission (Fukunaga et al., 2009; Yoon et al., 2014; Kovaleva et al., 2015; Pradel et al., 2019). Division by budding can thus be regarded as an uncommon feature in the class *Phycisphaerae* requiring further attention. This, however, exceeds the scope of this study, since we cannot exclude that strain KS4^T^ might even be capable to switch between both modes of cell division as recently shown in the proposed phylum *Saltatorellota* (Wiegand et al., 2019). The spherical cell shape and suspected mode of division by budding resembles members of the orders *Isosphaerales* or *Gemmatales*, while phylogenetically strain KS4^T^ is clearly a member of the class *Phycisphaerae*. Such findings might indicate that the mode of cell division is not necessarily a conserved feature in different classes within the phylum *Planctomycetes*. This is not entirely unlikely as the class *Phycisphaerae* is quite heterogeneous (see physiological and genomic comparison of strain KS4^T^ in the following section and Table 1). At this stage, it should also be kept in mind that the current closest relative of strain KS4^T^ has a 16S rRNA gene sequence similarity of only 88.7%, which is close to the family threshold of 86.5%.

**Table 1:** Phenotypic and genotypic features of strain KS4^T^ compared to closely related strains in the class *Phycisphaerae*.

Feature	KS4^T^	*P. mikurensis*	*A. agarilytica*	*T. mucosa*
Phenotypic features				
Shape	spherical	spherical	spherical	spherical
Diameter (μm)	0.63 ± 0.21	0.5–1.3	1.0–1.2	0.5–1.2
Color	white	pink	reddish-pink	white to rosy
Temperature range (optimum; °C)	15–30 (27)	10–30 (25–30)	20–30 (28)	20–56 (47–50)
pH range (optimum)	6.5–8.0 (7.5)	n.d.	6.0–8.0 (7.5)	4.5–8.5 (7.0–7.5)
ASW tolerance (optimum; %)	50–100 (75)	30–200 (70–100)	n.d.	n.d.
NaCl tolerance [% (w/v)]	up to 4	n.d.	up to 6	up to 3
Division	inconclusive	binary fission	binary fission	binary fission
Motility	yes	yes	yes	yes
Crateriform structures	n.o.	no	n.d.	n.d.
Fimbriae	yes	yes	n.d.	n.o.
Stalk	n.o.	no	n.d.	no
Holdfast structure	n.o.	n.d.	n.d.	n.d.
Genomic features				
Genome size (bp)	4,291,168	3,884,382	n.d.	n.d.
Plasmids	no	n.d.	n.d.	n.d.
G + C content (%)	48.7 ± 0	73.2 ± 2.3	63.0	53.0
Completeness (%)	94.83	94.83	n.d.	n.d.
Contamination (%)	0	0	n.d.	n.d.
Coding density (%)	84.2	88.6	n.d.	n.d.
Total genes	3,714	3,176	n.d.	n.d.
Genes/Mb	866	817	n.d.	n.d.
Giant genes (>15 kb)	0	0	n.d.	n.d.
Protein-coding genes	3,659	3,109	n.d.	n.d.
Protein-coding genes/Mb	853	800	n.d.	n.d.
Hypothetical proteins	1,499	1,442	n.d.	n.d.
tRNAs	45	59	n.d.	n.d.
16S rRNA genes	1	1	n.d.	n.d.

The genome analysis is based on GenBank accession numbers CP036425 (strain KS4^T^) and GCA_000284115 (Phycisphaera mikurensis). n.o., not observed; n.d., not determined.

### Analysis of Phenotypic Characteristics of Strain KS4^T^

**Figure 5:**
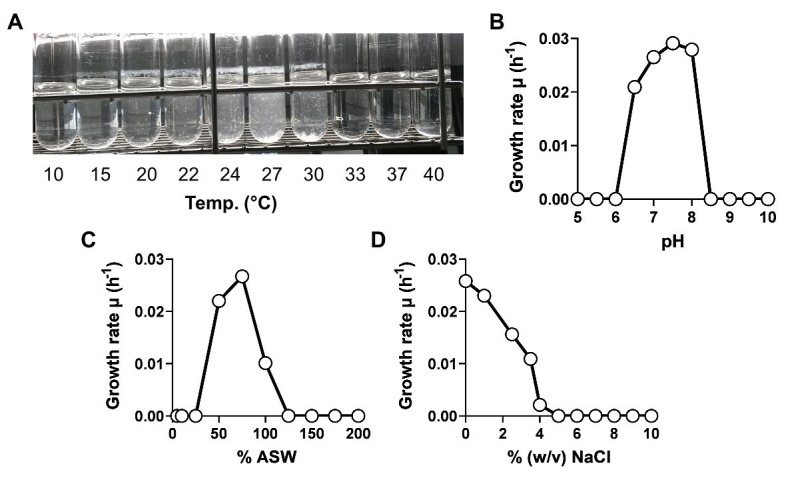
Physiological characterization of strain KS4^T^. Growth of strain KS4^T^ under different cultivation conditions was tested. Temperature **(A)**, pH **(B)**, artificial seawater **(C)**, and NaCl concentration **(D)** ranges enabling growth were analyzed by cultivation of the strain in M1H NAG ASW medium in biological triplicates. Due to the aggregation of cells at temperatures above and below the temperature optimum, no growth rates could be calculated for this experiment. The temperature optimum was determined by visual inspection of the cultivation tubes.

The physiology of strain KS4^T^ was analyzed during laboratory-scale cultivations in M1H NAG ASW medium, which was also used for the initial cultivation of the strain after isolation. Growth of strain KS4^T^ turned out to be planktonic during cultivation at the optimal temperature of 27°C, which allowed measurement of cell densities as the optical density at 600 nm (OD_600_). However, at temperatures below or above the optimal temperature, strain KS4^T^ formed larger aggregates in form of white flakes, which settled down in the cultivation tube (Figure 5A). In such cases, resuspension of cells and OD_600_ measurement was not possible. For determination of the temperature optimum, the tubes were thus inspected visually. All other experiments were conducted at 27°C, which allowed to measure cell densities and to calculate maximal growth rates. Strain KS4^T^ is able to grow over a temperature range of 15–30°C (optimum 27°C) and a pH range of 6.5–8.0 (optimum 7.5; Figures 5A,B). The maximal growth rate was calculated to be 0.029 h^−1^, which corresponds to a generation time of 24 h. Artificial seawater concentrations of 50–100% allowed for growth, with the highest growth rate observed at a concentration of 75% (Figure 5C). The strain tolerates a maximal NaCl concentration of 4% (w/v), but showed optimal growth in the absence of NaCl (Figure 5D). Strain KS4^T^ is heterotrophic, aerobic, mesophilic, and neutrophilic. Physiological features observed for different species in the class *Phycisphaerae* are quite heterogeneous. Strain KS4^T^ and the type strains of *P. mikurensis*, *A. agarilytica*, and *Tepidisphaera mucosa* cover a similar pH range and grow optimally at pH 7.5 (Table 1; Fukunaga et al., 2009; Yoon et al., 2014; Kovaleva et al., 2015). The first three strains have a similar temperature preference (25–28°C), while *T. mucosa* is thermophilic (growth optimum 47–50°C). Salt tolerance varies between 3 and 6% (w/v) NaCl and colony colors cover the entire range from white (unpigmented) to reddish-pink (Table 1).

### Analysis of Genome-Encoded Features of Strain KS4^T^

The comparison of genomic characteristics focused on *P. mikurensis* as the genomes of *A. agarilytica* and *T. mucosa* have not been sequenced. The genomes of *P. mikurensis* and strain KS4^T^ are similar in size (3.9–4.3 Mb) and also show similar numbers of protein-coding genes and coding densities (Table 1). Both genomes harbor a single copy of the 16S rRNA gene. The two genomes are clearly distinguishable by a different G + C content. Strain KS4^T^ has a moderate G + C content of 49%, whereas *P. mikurensis* features 73%, which is among the highest G + C contents found in bacteria (Hildebrand et al., 2010). We also analyzed the numbers of carbohydrate-active enzymes and genes/gene clusters putatively involved in secondary metabolite production based on the genomes of strain KS4^T^ and *P. mikurensis* NBRC102666^T^ (Table 2). A total number of 140–180 carbohydrate-active enzymes was found to be encoded in the genome of the two strains. The numbers do not reflect the genome size since strain KS4^T^ has a slightly larger genome, but 36 carbohydrate-enzymes less than *P. mikurensis*. About 43–45% of the enzymes fall within the family of glycosyl hydrolases and a similar number within the family of glycosyl transferases. The residual 10% are distributed among polysaccharide lyases, carbohydrate esterases, carbohydrate-binding module proteins and auxiliary proteins.

**Table 2:** Numbers of carbohydrate-active enzymes and secondary metabolite-associated gene clusters.

Feature	KS4^T^	*P. mikurensis*
Genome size (Mb)	4.29	3.88
Carbohydrate-active enzymes		
Glycoside hydrolase family	60	79
Glycosyl transferase family	62	77
Polysaccharide lyase family	3	2
Carbohydrate esterase family	4	3
Auxiliary activity family	1	0
Carbohydrate-binding module family	9	14
Total	**139**	**175**
Secondary metabolite-associated clusters		
Terpenoid	2	1
Type I PKS	0	0
Type II PKS	0	0
Type III PKS	0	1
Type I PKS-NRPS	0	0
NRPS	0	0
Bacteriocin	0	0
Ectoine	0	0
Resorcinol	0	0
Other	0	1
Total	**2**	**3**

The genome analysis is based on GenBank accession numbers CP036425 (strain KS4^T^) and GCA_000284115 (Phycisphaera mikurensis).

The number of secondary metabolism-related gene clusters typically correlates with the genome size. Not surprisingly, given the relatively small genomes of around 4 Mb compared to most of the other known members of the phylum *Planctomycetes*, the numbers of predicted gene clusters involved in secondary metabolite production are rather small. One to two identified clusters are related to terpenoid production. These are probably involved in carotenoid production in pigmented *P. mikurensis*. Since strain KS4^T^ is non-pigmented, the cluster may also be relevant for the synthesis of other terpenoids, e.g., hopanoids, as indicated by the presence of a squalene-hopene cyclase-encoding gene (locus KS4_02460) in strain KS4^T^. Except for a single putative type III polyketide synthase-encoding gene present in *P. mikurensis*, but absent in strain KS4^T^, both appear to lack PKSs and non-ribosomal peptide synthetases. Given that planctomycetes with larger genomes harbor up to 13 gene clusters associated with secondary metabolite production (Wiegand et al., 2020), such strains appear to be a more promising basis for discovery of bioactive molecules than the two here analyzed strains of the family *Phycisphaeraceae*.

Taken together, our analysis of physiological and genomic features of strain KS4^T^ supports the results of the phylogenetic inference, which together delineate the novel strain from known species and genera within the class *Phycisphaerae*. We thus conclude that strain KS4^T^ represents a novel species of a novel genus, for which we propose the name *Poriferisphaera corsica* gen. nov., sp. nov.

### Genus and Species Description

#### Description of *Poriferisphaera* Gen. Nov.

Po.ri.fe.ri.sphae’ra. N.L. neut. pl. n. *Porifera* name of a phylum (sponges); L. fem. n. *sphaera* a ball, sphere; N.L. fem. n. *Poriferisphaera* a spherical bacterium isolated from *Porifera*.

Members of the genus form spherical and motile cells. Cells are aerobic heterotrophs with a mesophilic and neutrophilic growth profile and lack crateriform structures, stalk structure, and holdfast structure. The G + C content of the genome is around 49%. The genus belongs to the family *Phycisphaeraceae*, order *Phycisphaerales*, class *Phycisphaerae*, phylum *Planctomycetes*. The type species of this genus is *Poriferisphaera corsica*.

#### Description of *Poriferisphaera corsica* Sp. Nov.

cor’si.ca. L. fem. adj. *corsica* corsican; corresponding to the origin of the strain from the Mediterranean island Corsica.

In addition to the genus characteristics, cells have an average diameter of 0.63 ± 0.21 μm and form aggregates. The cell surface is covered with outer membrane vesicles. Cells are aerobic and show asymmetric cell division. Cells of the type strain grow over a range of 15–30°C (optimum 27°C), pH 6.5–8.0 (optimum 7.5) and up to NaCl concentrations of 4% (w/v). Optimal growth was observed at 75% ASW, while amounts higher than in standard M1H NAG ASW (>100% ASW) or below 50% abolished growth. Colonies are white.

The type strain is KS4^T^ (=DSM 103958^T^ = LMG 29824^T^), isolated from the calcareous sponge *C. clathrus* close to the island Corsica, France, in September 2015. The genome size of the type strain is 4.29 Mb with a DNA G + C content of 48.7%.

## Data Availability Statement

The datasets presented in this study can be found in online repositories. The names of the repository/repositories and accession number(s) can be found at: https://www.ncbi.nlm.nih.gov/genbank/, CP036425, https://www.ncbi.nlm.nih.gov/genbank/, MK559978.

## Author Contributions

NK analyzed the cultivation data and wrote the article. SW and MJo performed the genomic and phylogenetic analyses. TK performed the amplicon sequencing. CB performed the light microscopic analysis together with TK. R-WM and FB led the sampling expedition and performed initial experiments in Corsica. OJ, PR, R-WM, and FB contributed to data analysis and preparation of the figures. AH and PR isolated the strain and performed the strain cultivation and deposition. MJe contributed to text preparation and revised the article. MR performed the electron microscopic analysis. CJ was involved in sampling and supervised the study. All authors contributed to the article and approved the submitted version.

### Conflict of Interest

The authors declare that the research was conducted in the absence of any commercial or financial relationships that could be construed as a potential conflict of interest.
